# “As a Young Pregnant Girl… The Challenges You Face”: Exploring the Intersection Between Mental Health and Sexual and Reproductive Health Amongst Adolescent Girls and Young Women in South Africa

**DOI:** 10.1007/s10461-020-02974-3

**Published:** 2020-07-18

**Authors:** Zoe Duby, Tracy McClinton Appollis, Kim Jonas, Kealeboga Maruping, Janan Dietrich, Ashleigh LoVette, Caroline Kuo, Lieve Vanleeuw, Catherine Mathews

**Affiliations:** 1 Health Systems Research Unit, South African Medical Research Council, Francie van Zijl Drive, Parow Valley, Tygerberg, Cape Town, South Africa; 2 Division of Social and Behavioural Sciences in the School of Public Health and Family Medicine, University of Cape Town, Cape Town, South Africa; 3 Adolescent Health Research Unit, Department of Psychiatry and Mental Health, University of Cape Town, Cape Town, South Africa; 4 Perinatal HIV Research Unit, School of Clinical Medicine, Faculty of Health Sciences, University of the Witwatersrand, Johannesburg, South Africa; 5 Department of Behavioral and Social Sciences, Brown University School of Public Health, Providence, RI USA

**Keywords:** Adolescent girls and young women, Depression, Suicide, Mental health, HIV, South Africa

## Abstract

In South Africa, adolescent girls and young women (AGYW) are at risk of poor mental health, HIV infection and early pregnancy. Poor mental health in AGYW is associated with increased sexual risk behaviours, and impeded HIV testing and care. Using in-depth interviews and focus group discussions, we explored subjective experiences of mental health and sexual and reproductive health (SRH) amongst 237 AGYW aged 15–24 years in five South African districts. Respondents shared narratives of stress, emotional isolation, feelings of depression, and suicidal ideation, interconnected with HIV, pregnancy and violence in relationships. Findings show that AGYW in South Africa face a range of mental health stressors and lack sufficient support, which intersect with SRH challenges to heighten their vulnerability. Framed within the syndemic theory, our findings suggest that South African AGYW’s vulnerability towards early pregnancy, HIV infection and poor mental health are bidirectional and interconnected. Considering the overlaps and interactions between mental health and SRH amongst AGYW, it is critical that mental health components are integrated into SRH interventions.

## Introduction

Poor mental health, including depressive disorders and stress, contributes significantly to the burden of disease in South Africa, and other parts of sub-Saharan Africa, and is also associated with negative sexual and reproductive health (SRH) outcomes for women, such as ‘unintended’ or early pregnancy, and increased risk behaviours for HIV [1–3]. Researchers in the field of women’s health have highlighted the need to further explore the syndemic interactions between psychosocial vulnerability, mental health, HIV infection, and poor SRH outcomes [4]. In South Africa’s HIV epidemic, the largest in the world, a quarter of all new infections occur amongst adolescent girls and young women (AGYW) aged 15–24, three times as high as their male counterparts [5]. As with HIV, South Africa also has high rates of teenage pregnancy; in 2016, 16% of females aged 15–18 years had begun childbearing [6]. AGYW in South Africa are more susceptible to depressive symptoms than their male counterparts, and are likely to remain under-diagnosed and untreated [7–9]. Thus, their vulnerability lies at a biological, social, and environmental nexus. The onset of depression in particular, but other mental health problems as well, can coincide with other developmental milestones such as sexual debut and escalated risks for HIV infection.

### Age and Socio-Economic Related Risk Factors

Estimates suggest that approximately three quarters of mental health comorbidities that affect adults across the life course emerge during adolescence and young adulthood [7]. Adolescents’ mental health status can have profound impacts on their future health, social, and economic circumstances as adults, particularly in contexts of poverty and vulnerability [10]. The development of poor mental health outcomes during this period is influenced by neurological, hormonal, and physical changes associated with puberty, combined with changes in adolescents’ social environments [11]. Evidence from South Africa and other countries in the sub-Saharan African region show that age-specific risk factors for depression and anxiety disorders include lower socio-economic status, lack of social capital and support, substance use, and exposure to violence and traumatic events [12]. Adolescents growing up in the context of socio-economically adverse communities are faced with a range of additional psychosocial and health risks that may evoke stress and negatively affect their mental health; these risks include exposure to HIV, substance use, violence, and other stressors [13]. Poverty has been shown to be associated with heightened vulnerability to experiencing poor mental health, including mood and anxiety disorders [13].

In addition to age-related factors and socio-economic factors, gender-related factors, including sexual and reproductive biology, also play a role in contributing to mental health risks. Adolescent pregnancy poses a significant mental health burden, predisposing AGYW to adverse mental health outcomes, with depression and anxiety being the most common [14]. In resource-deprived settings in sub-Saharan Africa, pregnancy amongst AGYW is associated with adverse mental health outcomes and psychosocial stresses including stigma and discrimination [14]. In the South African context, pregnancy may exacerbate existing social and contextual stressors, adding additional stressors such as interpersonal relationship challenges, regret around ‘unintended’ pregnancies, and depression [15]. Globally, suicide is the second leading cause of mortality among females aged 10–24 years; with low and middle income countries accounting for over 75% of global suicide deaths [11]. Rates of suicidal ideation, defined as the thought of killing oneself, are highest among adolescents on the African continent, with HIV as a contributing factor [11].

The syndemic theory of health refers to the clustering of risk factors, or co-occurring and intersecting epidemics embedded in the particular social context in which an individual is situated, which combine and interact to create vulnerability to health outcomes that are worse than any one risk factor alone would cause [16, 17]. By focusing on the ‘biosocial complex’, the interconnected and co-occurring health issues, as well as the social and environmental factors that promote and enhance negative health outcomes, the syndemic theory can help to explain the way in which risk behaviours which lead to negative SRH outcomes, namely HIV infection and ‘unintended’ pregnancy, are situated within co-occurring and interacting psychosocial health conditions, including psychological distress and poor mental health [9, 18, 19].

The immense physical, neurocognitive, mental, and social changes that occur during adolescence not only affect mental health, but also influence sexual behaviour; during this period of transitioning to adulthood, adolescents are at increased risk of HIV infection [18]. The association between depressive symptoms and decreased sexual agency and decision-making power in AGYW are compounded by low self-esteem; in turn these are associated with increased risk behaviours, including increased susceptibility to pressure to have sex, comfort seeking, condomless sex, transactional sex, trans-generational sex, substance use, and ‘unintended’ pregnancy [7, 8, 19–21]. Some of the mechanisms through which poor mental health symptoms influence sexual risk include substance use, maladaptive coping mechanisms to deal with stress, and impaired decision-making, indicating poor mental health as a prospective predictor of sexual risk [22]. Depressive symptoms in AGYW have also been correlated with a lack of ability to withstand social pressure, including peer pressure to engage in risky behaviours, a tendency to be more subservient and less assertive in sexual relationships, as well as with being more vulnerable to intimate partner violence and abuse [8, 19]. In addition to the links between depression and increased sexual risk taking, depression is also associated with impeded health seeking behaviour, including HIV testing [23].

Considering the overlaps and interactions between mental health and SRH amongst AGYW is critical. Greater insight into the lived subjective experience of depression and stress, and how these are linked to SRH outcomes is needed. There appears to be a gap in the literature pertaining to the ways in which mental health and psychosocial risks, including depression and stress, intersect and overlap with SRH related factors such as distress caused by ‘unintended’ pregnancy, material/emotional stressors of having a child, or social stigma [16]. Various studies explore depression amongst HIV positive women, but there has been little exploration of mental health issues that arise due to, or co-occur with, SRH outcomes. Although mental health was not an initial focus of the research, upon qualitative enquiry, the significance of poor mental health outcomes impacting on sexual and reproductive health practices emerged as a salient theme, warranting closer examination. We examined AGYW’s narratives and conceptualizations of their own mental health, that of peers, and of the surrounding emotional and psycho-social support context in order to explore the ways in which these factors might interact with sexual health outcomes.

## Methods

This research formed part of the HERStory study, which evaluated a comprehensive combination HIV prevention intervention for AGYW implemented in ten priority districts in South Africa from 2016 to 2019, funded by the Global Fund (https://www.samrc.ac.za/intramural-research-units/HealthSystems-HERStory). Included in the analysis for this paper are qualitative data from 63 in-depth interviews (IDIs) and 24 focus group discussions (FGDs) conducted between August 2018 and March 2019 in five South African districts, with a total of 237 AGYW aged 15–24 years. Of the 237 AGYW, 63 were from the Western Cape (WC), 50 from KwaZulu-Natal (KZN), 41 from Mpumalanga (MPU), 35 from the North West (NW), and 48 from the Eastern Cape (EC). Participant recruitment took place in selected schools and communities through liaising with school staff and/or intervention implementers in order to identify eligible participants, arrange interviews, and secure appropriate venues. A brief demographic questionnaire was also administered.

IDIs (20–40 min) and FGDs (40–90 min) were conducted in English, isiZulu, isiXhosa, seTswana, or siSwati, by one of two lead interviewers, accompanied by a research assistant, all female, and all of whom had received training on the study protocol, design, research tools, and human subject research ethics. Semi-structured topic guides comprised of open-ended questions and probes guided discussions. Included in the topic guides were questions relating to sources of social/emotional support; AGYW were asked who they talk to or seek support from when experiencing emotional/relationship/school/health/SRH concerns or challenges. Mental health was not a specific focus of the study but arose in response to these. The research team engaged in an on-going reflective process of note-taking and debriefing discussions, which formed part of the collaborative interpretation discussions and analysis process.

### Ethical Considerations

Informed consent was obtained from all participants 18 years and older. Written assent with written guardian consent was obtained for those younger than 18 years. Participants were provided with a ZAR 50.00 (approximately US$ 3.00) supermarket voucher, transport reimbursement, and refreshments. The study protocol and research tools were approved by the South African Medical Research Council Research Ethics Committee, and by the Associate Director for Science in the Center for Global Health in the Centers for Disease Control and Prevention. The research team received training on the study protocol and procedures for reporting and managing social harms and adverse events, as outlined in human subject research ethical guidelines***. ***During data collection, private-sector social workers were procured to assist with ensuring access to social support services for participants who needed psychosocial support.

### Data Preparation and Analysis

Audio recordings of IDIs and FGDs were transcribed verbatim into their original language, reviewed by the researcher who conducted the interviewer for accuracy, translated into English and re-reviewed by the interviewer. Data analysis followed a thematic approach, in which a pre-determined deductive codebook underwent cyclical review and refinement [24–26]. Collaborative interpretation by the research team, comprising the two interviewers who were also co-investigators, along with four other co-investigators, included individual data immersion and familiarisation, repeated deep readings of transcripts, documentation of reflective thoughts, and sharing growing insights about the research topic during regular team discussions. The codebook was entered into NVivo 12 software, which was used to organise and label relevant text from the transcripts. As concepts and themes emerged, the team collaboratively reviewed them, returning to the data, and refining themes. Weekly research meetings were held throughout the data collection and analysis phases allowing for team debriefing and examination of how thoughts and ideas were evolving as they engaged with the data. Three feedback workshops were held with 32 AGYW aged 15–24 at three of the study sites, some of whom had previously participated in IDIs and FGDs, and some who had not. The objective of these workshops was to review and discuss the preliminary analysis and interpretations, ensure accurate and appropriate interpretation of the data, clarify misunderstandings, and confirm findings and interpretations. During the workshops, the research team summarised and presented key themes and findings to the participants, who were then invited to give feedback, discuss their interpretation of the findings, and expand or elaborate on themes. Facilitated discussions on each theme were captured through notes and audio recordings, transcribed and reviewed, and included in the overall analysis.

## Findings

### Demographic Characteristics

Amongst the 237 AGYW respondents aged 15–24, the mean age was 17.4 years. Of these, 99% (N = 235/237) self-reported to have been assigned female at birth. Amongst the AGYW respondents, 97% (N = 227/234) self-identified their gender as female, with two identifying as transgender, and three as gender-variant. Most, 86% (N = 202/235) of AGYW self-identified as heterosexual/straight, 4% (N = 9) as homosexual/gay/lesbian, and 7% (N = 17) as bisexual. For reporting of language spoken at home, the top three languages were isiXhosa (39%, N = 92/237), isiZulu (25%, N = 59/237), and siSwati (15%, N = 35/237). Overall, 18% (N = 41/232) of the AGYW reported to have had a pregnancy.

Emergent themes in the qualitative data included AGYW narratives and perceptions of depression, stress, and suicide. In the accounts of AGYW, poor mental health, including depression and suicidal risk were linked to sexual/romantic relationship challenges, early pregnancy and child-bearing, parenting responsibilities, experiences of violence/abuse, HIV status, and lack of emotional support. Suicide risk emerged as a salient theme and was associated with discovery of pregnancy or an HIV positive status, low self-esteem, and a lack of anyone to trust or confide in. In general, AGYW voiced a need for increased access to support, and additional information on mental health.

The findings presented below are arranged into key thematic areas that emerged during analysis. Illustrative quotations are excerpts from English transcripts or translations; in brackets are details of the respondents’ site and sample group. In selected excerpts, original language terms/words have been included in italicised brackets for the purpose of illustrating the exact words/language used by participants relating to key concepts associated with mental health. The rationale for this is that often concepts such as "depression” have been framed in a universal/Western way, without attention to contextual specificity. Where qualitative research uses translations, there is a danger of the original meaning and concept getting lost in the translation process, as translators seek to find ‘equivalent’ terms [26].

### Suicidal Ideation

Suicidal ideation emerged as salient theme across provinces, despite there being no specific question probes relating to suicide. According to AGYW, issues such as self-harm and having suicidal tendencies were common amongst their peers. One participant expressed hesitancy using the diagnosis of ‘depression’, but described self-harming and suicidal ideation: “There are girls, I don’t want to say ‘depressed’, but who do things like self-harming, some attempt suicide” (15–18 years, EC). AGYW made links between low self-esteem and self-worth, and lacking a sense of belonging, with suicidal ideation: “Most girls… have a low self-esteem… feel as though they don’t belong in this world. That’s why people commit suicide*.* I used to have that… mentality… suicidal thoughts because of people” (19–24 years, NW).

Illustrating the link between SRH and mental health, feelings of emotional isolation leading to suicidal ideation were exacerbated in the case of HIV positive or pregnant AGYW who feel unable to access support: “This thing of suicide is becoming popular now, even here at school… especially when girls are pregnant or HIV positive, because they can’t share it with anyone, they don’t trust anyone” (15–18 years, WC). The sense of having no one to trust or confide in, and seek emotional support from, resulted in AGYW feeling emotionally isolated, fostering suicidal ideation: “We don’t share our sexual and personal life things… We keep it to ourselves, then some of us commit suicide *(sizigcinia kuthi, abanye bethu ke baphela sebezibulala)”* (15–18 years, WC, isiXhosa). Suicide was linked to feelings of isolation after an HIV positive diagnosis: “(When) the nurse told her that is she is HIV positive, she didn’t know who to tell… so she took a rope and hanged herself because she had no one to talk to” (15–18 years, WC).

The discovery of being pregnant was also described as a difficult emotional event. AGYW in the older age group, 19–24 years, described personal experiences with suicidal ideation in this situation: “When I found out I was pregnant… that was very difficult, I even thought about suicide… it was tough *(kwabanzima kakhulu, ngangicabanga ngisho ukuyibulala, ya kwaku tough)”* (19–24 years, KZN, isiZulu). Additional links between mental health and SRH were apparent in the narratives of suicidal ideation in relation to the stress of teenage pregnancy, compounded by fear of HIV: “As a young pregnant girl… the challenges you face… maybe you will find out that he (baby’s father) is HIV-positive… Those are challenges that can be a problem and you end up committing suicide… A better solution is to kill yourself *(Yizona ngqinamba lezo ezingaba inkinga ugcine usu… usuzibula… i solutions kuncono ukuthi uzibulale)”* (19–24 years, KZN, isiZulu).

Respondents suggested that due to social stigma attached to teenage pregnancy, pregnant AGYW fear being judged and gossiped about: “Pregnant girls feel sad… some even contemplate suicide *(azive efuna ukuzibulala)…* because of hearing unpleasant things about their life being spoken by other people. (15–18 years, WC, isiXhosa); “Pregnant girls are teased, and then they drop out of school, they don't finish… here at school… we gossip about each other in the toilets” (15–18 years, WC). Parents’ attitudes towards their daughters’ romantic and sexual behaviour prevented AGYW from accessing support: “Like most girls, I got pregnant at an early age. Some girls resort to committing suicide *(ezibulala)* or just run away from home because they cannot face their parents” (15–18 years, WC, isiXhosa). Getting involved in transactional relationships, compounded by a sense of shame and fear of social judgement, also led to depression and suicidal ideation: “Most girls in the community, they get into those (transactional) relationships, to a point that it damages them… they end up being depressed*…* ‘Why are you doing this and that to me in front of people?’… they end up like that and they end up trying to commit suicide… ‘he embarrassed me in front of people, tomorrow how will people look at me?’” (19–24 years, NW).

### Stress

The emotional ‘burden’ of teenage pregnancy was described as a key contributing factor to poor mental health: “They say having a child is a good thing, but as a teenager it is a burden, it’s difficult to cope” (15–18 years, EC). Financial, material and relationship insecurity added stress to pregnancy: “the baby’s father has denied the baby, there will be stress of how you are going to support the baby, because the (social) grant is not enough” (19–24 years, KZN). Those AGYW who had experienced unexpected discovery of pregnancies described their stress related to being rejected by families, kicked out of school or from home. One participant described her concerns after finding out she was pregnant in Grade 10: “I was confused and didn’t know what to do… (I told my boyfriend) my dad is strict… I will be chased away from home” (19–24 years, KZN). Those AGYW who became pregnant with casual sex partners, or who were not in committed relationships described the stress and unhappiness they experienced. One young woman described how she wanted to terminate her pregnancy but was told it was already too late to do so, and how this unwanted pregnancy caused her stress: “I had stress… I only realised when I was 4 months 2 days that I was pregnant… if I had realised this earlier, I was going to do an abortion… then I asked the doctor ‘Is there any other way I can do an abortion?’… He then said ‘It’s either you die… I will not allow you to risk an abortion’…[sigh] I was not ready to have a child at that time… I knew how my situation was… the guy I was dating, I was just dating him for fun. I did not see myself having a child with him, or to have future with him… that was why I was going to abort this baby… I did not want the child… everything failed… I did not eat, I had stress… the one who impregnated me was staying in a shack” (19–24 years, NW).

A lack of emotional support from partners/fathers of children also contributed to stress and depression amongst young mothers: “Where does the stress go? …to me… I’m always watching this child, he cries the whole day and I don’t know why… I’m holding him, gave him his bottle, he continues to cry, I don’t know why he is crying… you call him (baby’s father)… (but) he doesn’t take any action… I become depressed and it affects the child” (19–24 years, NW). Being a single parent was described as difficult and stressful: “If you are a single mother, there is nothing nice… (you) have love for your baby but that’s it. Everything else is not nice… It’s difficult to raise the child” (19–24 years, NW). The feeling that former dreams and aspirations for the future were shattered by unexpected pregnancies heightened feelings of hopelessness and depression: “It’s not going to be dark forever, things will be right… but what I can say? …to be pregnant unexpectedly is not good at all… life is not good… Especially if… you had plans and maybe life does not go the way you had planned… I am speechless… for me now, life is not good… it’s not good… tough times…” (19–24 years, NW).

### Emotional Support

Lacking a supportive social environment negatively impacted on mental health and self-esteem: “When people are discouraging me… I get very sad… I’m trying… I’m telling them that… and they say ‘You cannot do that… you’re weak’… it makes me angry, but… I don’t defend myself” (19–24 years, NW). Some AGYW suggested that they tried to cope without sharing their problems with anyone: “I keep my problems to myself… I talk to no one*…* I keep to myself and own it, I don’t make my problem someone else’s… if ever I have something troubling me I will keep it to myself… eventually I will be fine *(ndizade ndibe right)”* (19–24 years, EC, isiXhosa). The lack of emotional support for dealing with traumatic life events, including grief over the death of a loved one, was present in AGYW narratives: “When I think about something that happened in the past my heart becomes sore *(intliziyo yam ibabuhlungu)…* (like) when I think about my mother… she passed away… There is nobody (I talk to at home)… I don’t feel free talking to them… I don’t speak to anyone at school (either)” (15–18 years, EC, isiXhosa). A minority of AGYW were vocal about receiving emotional support at home: “I know I am loved at home and they show me that they love me because they care for me and stuff” (19–24 years, EC).

Sexual and romantic relationships with violent and controlling partners were also described by some of the AGYW, who ended up living in a state of fear: “If I have made friends… and we want to go out as girls, then he (boyfriend) will refuse and beat you. Even when you make a minor mistake… he will beat you, and you end up afraid… you now live in fear… When happy, it doesn’t last for long… sometimes he will take out his anger on you even when you did nothing… but you continue to love him even when friends try to talk some sense to you, but you will continue staying and loving him because you are afraid of him and not at liberty to do your own things” (19–24 years, MPU). Refusal to have sex with a partner also led to violence: “sometimes, it happens that he wants to sleep with you, and you don’t want to, then he gets angry and he beats you” (19–24 years, KZN). Those AGYW who had experienced intimate partner violence explained their reluctance to disclose to her family and friends: “In most times, you keep quiet and when they ask you ‘what happened, why are you hurt?’ you just tell them that you got hurt, you turned and bumped into a wall” (19–24 years, KZN). Experiencing violence negatively impacted AGYW self-esteem and self-worth: “It has to do with how you perceive yourself, he sees me as not good enough then maybe you will find that boyfriend that hits you, he is the one that you want to stay with because you think where else will you find another boyfriend? …when he hits you that means this person does not see any value on you he beats you, abuses you… physically you will be injured obviously because that will hurt you… and she will think that she is not good enough” (15–18 years, EC). Pregnancy increased AGYW dependence on partners, even when they are violent: “My friend is pregnant… (her boyfriend) beats her… In her pregnancy the guy did not care for her and he was beating her saying the child is not his” (19–24 years, EC).

## Discussion

Our study did not initially set out to examine mental health amongst AGYW, but narratives around depression, stress and suicide became salient, as did evidence of their interconnection with sexual and reproductive health. Feelings of stress, anxiety and not being able to cope, even to the point of suicide ideation, were associated with HIV status, unexpected discovery of pregnancy, and parenting responsibilities. Violence in relationships, a lack of emotional support from family and partners, and financial insecurity interact to exacerbate AGYW vulnerability to poor mental health and SRH outcomes.

AGYW in our study who had been pregnant, shared narratives of negative emotions they had experienced on discovering their pregnancy, leading to depression and suicidal ideation. The social causation hypothesis theory posits that stressful circumstances or events increase an individual’s susceptibility to manifesting or experiencing mental health problems [13]. It would therefore make sense that the emotional aspects related to the discovery of an unexpected pregnancy or an HIV positive status would act as a stressor and have potentially negative mental health outcomes [16]. It is likely that after encountering a stressor, adolescents will experience stress and symptoms of depression and anxiety [13]. The stress related to the discovery of an unexpected pregnancy is compounded by the shame and social stigmatisation of teenage pregnancy, and the ensuring social isolation from family and community increases the risk for psychological distress [7]. The framing of pregnancy during adolescence as a social problem means that pregnant teens receive limited social support, which in turn is linked to poor mental health outcomes [2]. The stress related to the discovery of an unexpected pregnancy is heightened further in the case of a dual discovery of being HIV positive [27]. The presence of depression, anxiety, and post-traumatic stress disorder in HIV-positive individuals is related to diagnosis and disclosure, and HIV-positive women experiencing ‘unintended’ pregnancy are at high risk for antenatal depression [27, 28].

AGYW in our study described a worrying trend of suicidal ideation. Thoughts about suicide narrated by AGYW were related to unexpected discovery of pregnancy and its consequences, HIV diagnosis, and feelings of emotional isolation. Suicide was described as “the best solution” to situations of stress created by the discovery of unexpected discovery of pregnancy or HIV positive status, lack of material/financial or emotional support for young mothers, and feelings of victimisation as a result of gossip or judgement. Adolescents faced with multiple stressors may experience a sense of being overwhelmed and unable to cope, and view suicide as an escape [13]. AGYW in our study associated low self-esteem and low self-worth with depression and suicidal ideation. Negative self-cognitions and low self-worth are associated with depression, and positive self-esteem is a critical component of emotional well-being [29]. Adolescents’ ability to manage their stress symptoms or address the stressor they encounter may be pivotal to protecting their mental health state, as it may buffer the impact of experienced stress on mental health [13]. Additionally, self-esteem and social support are amongst the ‘protective assets’ associated with improved SRH outcomes [30]. In our study, AGYW expressed feeling emotionally isolated, lacking people who they felt they could trust and confide in without fear of judgement or recrimination. Emotional isolation and lack of support, especially when faced with stressors such as the discovery of an unexpected pregnancy or an HIV diagnosis, negatively impacts mental health. The emotionally distressing aspects of unexpected discovery of pregnancy, or an HIV diagnosis, combined with a lack of social support, contribute to the high rates of depression amongst AGYW [16].

AGYW in our study described additional stress related to teenage pregnancy and child-bearing relating to concern around the ability to support a baby financially, especially when there was a lack of material and/or emotional support from the father of the child. AGYW in South Africa, particularly those in resource-constrained or violent households, face a variety of personal and structural challenges, linked to disempowerment and psychological distress more broadly; an ‘unintended’ pregnancy can compound pre-existing social and economic vulnerabilities, and result in heightened feelings of stress and unhappiness [31]. Socio-economic disadvantage compounds other stressors in an adolescents’ life, and when co-occurring with pregnancy or HIV, can lead to poor mental health outcomes and a lack of utilisation of health care services [2, 13].

One limitation of this study was that the framing and concept of ‘unintended’ pregnancy was not investigated in more depth. For this reason, we avoid using the term ‘unintended’, and instead use the words of AGYW respondents themselves when describing their unexpected discovery of a pregnancy, rather than objectively categorising the pregnancies as ‘unintended’. In addition, social desirability bias relating to the stigma and shame around mental health may have resulted in a lack of disclosure from AGYW regarding their own feelings of depression or suicide ideation.

## Conclusions and Recommendations

Interpreting our findings within a syndemic theory framework is helpful, in order to describe the integration of socio-cultural, psychological, and physiological factors that combine to shape AGYW vulnerabilities and experiences [32]. Our findings suggest that South African adolescent girls and young women’s vulnerability towards early pregnancy, HIV infection and poor mental health are bidirectional and interconnected. The social context in which South African AGYW are situated, as described by respondents in our study, is characterised by a lack of social support, economic insecurity, and stigma, and serves to exacerbate the gendered and age-related vulnerabilities of this population. This interaction of socio-cultural, economic, structural, gendered, age-related and biological factors increase South African AGYW’s heightened risk of negative SRH outcomes, co-occurring with psychological distress and poor mental health [33].

### Integration of Mental Health into SRH Services for AGYW

In line with the syndemic theory suggesting synergistic interactions between epidemics, and the interconnectedness and clustering of psychosocial conditions such as ‘unintended’ pregnancies, psychosocial distress, and HIV infection in AGYW, there is a need for comprehensive HIV prevention programming inclusive of mental health support [34]. It is clear that interventions aiming to reduce rates of teenage pregnancy and reduce HIV acquisition amongst AGYW in South Africa, need to incorporate mental health components [35]. Recommendations have been made for integrating mental health care into care for patients with chronic non-communicable diseases, as well as communicable diseases such as HIV [10], but few recommendations for integrating mental health into SRH delivery exist [36]. The links between mental health, HIV status, and ‘unintended’ pregnancy, exacerbate the need to strengthen the integration of routine mental health screening in SRH and HIV programming in order to enhance the health outcomes amongst AGYW [12]. Addressing underlying mental health risks may be an important additional strategy to promote sexual risk reduction, and behavioural interventions which are able to improve mental health are also more effective in preventing negative sexual health outcomes such as HIV infection [22].

### Mental Health Screening Included in SRH Services

The indication of a dual burden of psychological distress and sexual risk behaviours suggests that screening for mental health disorders should be integrated into SRH services [22]. Despite the evidence of intersecting epidemics, mental health screening is not standard in HIV prevention and care settings and has not been added to the HIV care cascade. Combination interventions inclusive of psychological and behavioural components may be able to achieve greater reductions in sexual risk behaviour among adolescents, as incorporating psychological health interventions appears to be a critical part of any comprehensive strategy for mitigating HIV risk [21]. Mental health services targeted at AGYW, especially those that are HIV positive and/or pregnant, need to be integrated into SRH services, especially those that aim to be “youth-friendly”; prevention, diagnosis and management of depressive symptoms should also be included in the package of comprehensive services [14, 19]. Early mental health screening could help catch AGYW who might not yet be diagnostically clinical depressed. Given the evidence, it is likely that AGYW have overlapping epidemics that are clinically significant. Practical recommendations for improving mental health care delivery to AGYW include improving mental health, advocacy, decentralization of services, task-shifting and on-the-job training [12, 37].

### Contextually Relevant Interventions

The way in which mental health issues, such as stress and depression, are defined and conceptualised differs across settings and socio-cultural contexts, and interventions needs to be contextually relevant [1]. In addition to contextual and conceptual equivalence, linguistic equivalence of terms related to mental health should be taken into account. The words “depression” and “anxiety” do not have direct equivalents in some South African languages, with psychological distress, including depression, described behaviourally rather than cognitively, and expressed and/or experienced somatically, or situated within the relational domain [29, 38]. In addition, the term “unintended pregnancy” is problematic, with traditional measurements dichotomously classifying pregnancies as intended or not, based on a woman’s intentions before she became pregnant [39, 40]. “Unplanned pregnancies” likewise have been defined as pregnancies which occur when a woman is using contraception or did not desire pregnancy as an outcome [39]. The key problem with these binary classifications of pregnancies as unintended/intended, planned/unplanned, is that they fail to consider the complexity of intention, motivation and desire [40]. The construct of ‘pregnancy acceptability’ may be a more useful way to taking into account the complexity of pregnancy intentions, in the context of women’s lived experiences, emotions, relationships, and socio-economic circumstances [40]. A woman’s emotions, levels of preparedness, and acceptance of a pregnancy are likely to change in reaction to external factors, which in turn influence health outcomes [40]. The biomedical/clinical understandings and definitions of “depression” and “unintended pregnancy” may not always capture an individual’s subjective experiences and articulation of these states [41], and in order for any intervention to be successful, there is a need to be sensitive and reflective of the reality of people’s lives, with an understanding of the language that AGYW use to describe their lived experiences of depression and pregnancy, which is why qualitative research such as the findings we present here, is much needed [40]. Indeed, AGYW in South Africa construct “depression” and pregnancy is a complex phenomenon manifesting in a variety of emotions, thoughts, and behaviours; finding ways to surface contextually congruent understandings of sexual and reproductive health, and mental health can inform the development of interventions that are contextually and population relevant [29]. Mental health and SRH interventions and services need to be contextually appropriate and reflective of the reality of people’s lives. Screening tools need to take into account the diversity of understandings of emotional suffering and distress, using appropriate terms, language and concepts.

It is evident that AGYW in South Africa face substantial social adversities and related mental health challenges due to a range of SRH, social, economic, environmental, physiological and interpersonal factors. Building on previous research that has found associations between depressive symptoms and psychological distress related to pregnancy, combined with a lack of social support amongst South African women [16], our findings provide rich descriptive data on the lived reality of the interconnected psychosocial risks including stress, emotional isolation, feelings of depression and suicidal ideation, with ‘unintended’ pregnancy and HIV that AGYW in South Africa face, from their own perspectives. Framing these interconnections within the syndemic framework can help to inform interventions that seek to address AGYW risk. As psychological distress is associated with increased risk behaviours, it is critical that efforts to address early pregnancy and HIV infection amongst AGYW incorporate mental health components. Interventions to improve emotional wellbeing and coping mechanisms for AGYW are needed in order to improve sexual and reproductive health outcomes; indeed, in a context where HIV, STIs, early pregnancy are common, it is all the more important to have such interventions integrated into SRH services and part of large-scale programmes for AGYW. Understanding the context of mental health is crucial in order to design and implement effective mental health programming, and to provide appropriate psycho-social support to young women, and in turn, address sexual and reproductive health challenges.

## References

[1] Kuo C, LoVette A, Stein DJ, Cluver LD, Brown LK, Atujuna M (2018). Building resilient families: developing family interventions for preventing adolescent depression and HIV in low resource settings. Transcult Psychiatry.

[2] Hill LM, Maman S, Groves AK, Moodley D (2015). Social support among HIV-positive and HIV-negative adolescents in Umlazi, South Africa: changes in family and partner relationships during pregnancy and the postpartum period. BMC Pregnancy and Childbirth.

[3] Gouda HN, Charlson F, Sorsdahl K, Ahmadzada S, Ferrari AJ, Erskine H (2019). Burden of non-communicable diseases in sub-Saharan Africa, 1990–2017: results from the Global Burden of Disease Study 2017. Lancet Glob Health.

[4] Zachek CM, Coelho LE, Domingues RMSM, Clark JL, De Boni RB, Luz PM (2019). The intersection of HIV, social vulnerability, and reproductive health: analysis of women living with HIV in Rio de Janeiro, Brazil from 1996 to 2016. AIDS Behav.

[5] Simbayi LC, Zuma K, Zungu N, Moyo S, Marinda E, Jooste S (2019). South African national HIV prevalence, incidence, behaviour and Communication Survey, 2017.

[6] National Department of Health, Statistics South Africa, South African Medical Research Council, ICF. South Africa Demographic and Health Survey 2016. Pretoria: National Department of Health; 2019

[7] Barhafumwa B, Dietrich J, Closson K, Samji H, Cescon A, Nkala B (2016). High prevalence of depression symptomology among adolescents in Soweto, South Africa associated with being female and cofactors relating to HIV transmission. Vulnerable Child Youth Stud.

[8] Fernander AF, Flisher AJ, King G, Noubary F, Lombard C, Price M (2006). Gender differences in depression and smoking among youth in Cape Town, South Africa. Ethn Dis.

[9] Sikkema KJ, Watt MH, Meade CS, Ranby KW, Kalichman SC, Skinner D (2011). Mental health and HIV sexual risk behavior among patrons of alcohol serving venues in Cape Town, South Africa. JAIDS J Acquir Immune Defic Syndr.

[10] Docrat S, Besada D, Lund C (2019). An evaluation of the health system costs of mental health services and programmes in South Africa.

[11] Casale M, Boyes M, Pantelic M, Toska E, Cluver L (2019). Suicidal thoughts and behaviour among South African adolescents living with HIV_ Can social support buffer the impact of stigma?. J Affect Disord.

[12] Kuringe E, Materu J, Nyato D, Majani E, Ngeni F, Shao A (2019). Prevalence and correlates of depression and anxiety symptoms among out-of-school adolescent girls and young women in Tanzania: a cross-sectional study. PLoS ONE.

[13] Harrison C, Loxton H, Somhlaba NZ (2019). Stress and coping: considering the influence of psychological strengths on the mental health of at-risk South African adolescents. Child Care Pract.

[14] Osok J, Kigamwa P, Vander Stoep A, Huang K-Y, Kumar M (2018). Depression and its psychosocial risk factors in pregnant Kenyan adolescents: a cross- sectional study in a community health Centre of Nairobi. BMC Psychiatry.

[15] Watt MH, Eaton LA, Choi KW, Velloza J, Kalichman SC, Skinner D (2014). “It's better for me to drink, at least the stress is going away”: perspectives on alcohol use during pregnancy among South African women attending drinking establishments. Soc Sci Med.

[16] Choi KW, Smit JA, Coleman JN, Mosery N, Bangsberg DR, Safren SA (2019). Mapping a syndemic of psychosocial risks during pregnancy using network analysis. Int J Behav Med.

[17] Illangasekare S, Burke J, Chander G, Gielen A (2013). The syndemic effects of intimate partner violence, HIV/AIDS, and substance abuse on depression among low-income urban women. J Urban Health.

[18] Mpondo F, Ruiter RAC, Schaafsma D, van den Borne B, Reddy PS (2018). Understanding the role played by parents, culture and the school curriculum in socializing young women on sexual health issues in rural South African communities. J Soc Asp HIV/AIDS.

[19] Nduna M, Jewkes RK, Dunkle KL, Shai NPJ, Colman I (2010). Associations between depressive symptoms, sexual behaviour and relationship characteristics: a prospective cohort study of young women and men in the Eastern Cape, South Africa. J Int AIDS Soc.

[20] Pengpid S, Peltzer K, Skaal L (2013). Mental health and HIV sexual risk behaviour among University of Limpopo students. S Afr J Psychiatry.

[21] Thurman TR, Kidman R, Carton TW, Chiroro P (2016). Psychological and behavioral interventions to reduce HIV risk: evidence from a randomized control trial among orphaned and vulnerable adolescents in South Africa. AIDS Care.

[22] Hill LM, Maman S, Kilonzo MN, Kajula LJ (2017). Anxiety and depression strongly associated with sexual risk behaviors among networks of young men in Dar es Salaam, Tanzania. AIDS Care.

[23] Rane MS, Hong T, Govere S, Thulare H, Moosa M-Y, Celum C (2018). Depression and anxiety as risk factors for delayed care-seeking behavior in human immunodeficiency virus-infected individuals in South Africa. Clin Infect Dis.

[24] Bradley EH, Curry LA, Devers KJ (2007). Qualitative data analysis for health services research: developing taxonomy, themes, and theory. Health Serv Res.

[25] Nowell LS, Norris JM, White DE, Moules NJ (2017). Thematic analysis: striving to meet the trustworthiness criteria. Int J Qual Methods.

[26] Vaismoradi M, Jones J, Turunen H, Snelgrove S (2016). Theme development in qualitative content analysis and thematic analysis. JNEP.

[27] Crankshaw TL, Voce A, King RL, Giddy J, Sheon NM, Butler LM (2014). Double disclosure bind: complexities of communicating an hiv diagnosis in the context of unintended pregnancy in Durban. S Afr AIDS Behav.

[28] Collins PY, Holman AR, Freeman MC, Patel V (2006). What is the relevance of mental health to HIV/AIDS care and treatment programs in developing countries? A systematic review. AIDS.

[29] Meyer K, Kruger L-M (2015). “You get angry inside yourself”: low-income adolescent South African girls’ subjective experience of depression. Soc Work Maatsk Werk.

[30] Plourde KF, Ippoliti NB, Nanda G, McCarraher DR (2017). Mentoring interventions and the impact of protective assets on the reproductive health of adolescent girls and young women. JAH.

[31] Lewinsohn R, Crankshaw T, Tomlinson M, Gibbs A, Butler L, Smit J (2018). “This baby came up and then he said, ‘I give up!’: the interplay between unintended pregnancy, sexual partnership dynamics and social support and the impact on women’s well-being in KwaZulu-Natal, South Africa. Midwifery.

[32] Mendenhall E (2015). Beyond comorbidity: a critical perspective of syndemic depression and diabetes in cross-cultural contexts. Med Anthropol Q.

[33] Singer M, Bulled N, Ostrach B, Mendenhall E (2017). Syndemics and the biosocial conception of health. Lancet.

[34] Sileo KM, Kershaw TS, Callands TA (2019). A syndemic of psychosocial and mental health problems in Liberia: examining the link to transactional sex among young pregnant women. Glob Public Health.

[35] Docrat S, Besada D, Cleary S, Daviaud E, Lund C (2019). Mental health system costs, resources and constraints in South Africa: a national survey. Health Policy Plan.

[36] Schneider M, Baron E, Breuer E, Docrat S, Honikman S, Onah M (2016). Integrating mental health into South Africa’s health system: current status and way forward. S Afr Health Rev.

[37] Reddy S, James S, Sewpaul R, Sifunda S, Ellahebokus A, Kambaran N, et al. Umthente Uhlaba Usamila—The 3rd South African National Youth Risk Behaviour Survey 2011. Cape Town; 2013

[38] Sweetland AC, Belkin GS, Verdeli H (2014). Measuring depression and anxiety in sub-Saharan Africa. Depress Anxiety.

[39] Santelli J, Rochat R, Hatfield-Timajchy K, Gilbert BC, Curtis K, Cabral R (2003). The measurement and meaning of unintended pregnancy. Perspect Sex Reprod Health.

[40] Gomez AM, Arteaga S, Ingraham N, Arcara J, Villaseñor E (2018). It's not planned, but is it okay? The acceptability of unplanned pregnancy among young people. Women's Health Issues.

[41] Dukas C (2014). A feminist phenomenological description of depression in low-income South African women.

